# Sequential biventricular pacing improves regional contractility, longitudinal function and dyssynchrony in patients with heart failure and prolonged QRS

**DOI:** 10.1186/1476-7120-8-12

**Published:** 2010-04-12

**Authors:** Magnus Edner, Margareta Ring, Tooomas Särev

**Affiliations:** 1 Karolinska Institutet, Department of Clinical Sciences, Danderyd Hospital, Division of Cardiovascular Medicine, Stockholm, Sweden; 2 Department of Molecular Medicine and Surgery, Karolinska Institutet, Stockholm, Sweden

## Abstract

**Aims:**

Biventricular pacing (BiP) is an effective treatment in systolic heart failure (HF) patients with prolonged QRS. However, approximately 35% of the patients receiving BiP are classified as non-responders. The aim of this study is to evaluate the acute effects of VV-optimization on systolic heart function.

**Methods:**

Twenty-one HF patients aged 72 (46-88) years, QRS 154 (120-190) ms, were studied with echocardiography, Tissue Doppler Imaging (TDI) and 3D-echo the first day after receiving a BiP device. TDI was performed; during simultaneous pacing (LV-lead pacing 4 ms before the RV-lead) and during sequential pacing (LV 20 and 40 ms before RV and RV 20 and 40 ms before LV-lead pacing). Systolic heart function was studied by tissue tracking (TT) for longitudinal function and systolic maximal velocity (SMV) for regional contractility and signs of dyssynchrony assessed by time-delays standard deviation of aortic valve opening to SMV, AVO-SMV/SD and tissue synchronization imaging (TSI).

**Results:**

The TT mean value preoperatively was 4,2 ± 1,5 and increased at simultaneous pacing to 5,0 ± 1,2 mm (p < 0,05), and at best VV-interval to 5,4 ± 1,2 (p < 0,001). Simultaneous pacing achieved better TT distance compared with preoperative in 16 patients (76%). However, it was still higher after VV-optimization in 12 patients 57%. Corresponding figures for SMV were 3,0 ± 0,7, 3,5 ± 0,8 (p < 0,01), and 3,6 ± 0,8 (p < 0,001). Also dyssynchrony improved.

**Conclusions:**

VV-optimization in the acute phase improves systolic heart function more than simultaneous BiP pacing. Long-term effects should be evaluated in prospective randomized trials.

## Introduction

Chronic heart failure (CHF) is perhaps the most common reason for in-hospital health care utilization and costs are very high. Despite drugs such as RAAS-blockers, beta-blockers, diuretics and aldosterone-antagonists which all have positive prognostic effects these patients suffer from poor quality of life (QoL) and a readmission rate as high as 30% within 90 days [1-5].

Cardiac Resynchronization Therapy (CRT) is established as an effective treatment in systolic heart failure (HF) patients with prolonged QRS being in functional class (NYHA) III-IV despite optimal medical treatment. CRT has been shown to improve heart function, QoL, morbidity and mortality [6-10]. However, approximately 30-40% of the patients receiving a CRT pacemaker do not respond as expected. There are several possible explanations to this such as non-viable myocardium, wrong indication, suboptimal LV lead location and one reason might also be suboptimal programming of the CRT device.

When giving effective drugs as RAAS- and beta-blockers it is obvious that dosages should be individualized and slowly optimized to achieve best effects and to avoid adverse effects. In CRT, however, it seems uncommon to individualize and optimize programming of the device and there are so far surprisingly few long-term randomized studies on this issue. Patients with CHF in NYHA-class III or IV have very high one- year mortality, 30-50%, which should make attempts to optimize the CRT device high priority. In the land-mark study CARE-HF [10], AV-delay was optimized, according to protocol, before discharge, after 3 months and every 6 months thereafter. This is today the recommendation by the American society of echocardiography [11].

The aim of this study was to evaluate the effect of VV-optimization during the first day after implantation of a CRT device on systolic heart function and signs of dyssynchrony.

## Methods and patients

### Study Population

We enrolled 21 consecutive heart failure patients with standard criteria for biventricular pacemaker treatment. All patients had reduced systolic left ventricular (LV) function with an ejection fraction (EF) below 35%, QRS - duration >120 ms and New York Heart Association (NYHA) function class III-IV despite optimal medical treatment. Twenty-one patients were on ACEi/ARB:s, 21 on diuretics and 19 were on beta blockers. Mean age was 72 (range 46-88) years and 18 patients were of male gender (Table 1). Exclusion criteria were artrial fibrillation. The study adhered to the Declaration of Helsinki and all patients gave informed consent. The study was approved by the Regional Ethical Review Board.

**Table 1:** Baseline characteristics of the patients (n = 21).

Variable	HF patients
Age (years)	72 ± 11 (46-88)
Gender (male %)	90
Heart Rate, bpm	67 ± 18 (range 35-129)
QRS-width, ms	154 ± 21 (120-190)
Function Class, NYHA (n)	
III	19
IV	2
Previous Myocardial Infarction	11
Hypertension	3
Cardiomyopathy	4
Diabetes	3
ACE/ARB	21
Diuretics	21
Beta-blockers	19
Spironolactone	15

Mean ± SD. HF= heart failure, NYHA = New York Heart Association, ACE = Angiotensin Converter Enzyme Inhibitor, ARB = Angiotensin Receptor Blocker.

### Echocardiography and Tissue Doppler Imaging

The patients were examined in the left lateral position and Tissue Doppler Imaging (TDI) echocardiography was acquired in three apical views using a vivid 7 or 5 (the later for 3-D echocardiography only) system (Vingmed, Hortem, Norway), with a 2.5 MHz probe. Three consecutive beats were registered and mean values were used for further analysis. Analyses were made off-line (Echo-Pac software, Hortem, Norway). Systole was defined as the ejection time from the aortic valve opening (AVO) to the aortic valve closure (AVC). The 16 segments LV-model of the American Society of Echocardiography was used for orientation [12]. Base and mid LV-segments, total of 12, were used together with base and mid RV-segments in the 4-chamber view. The LV enddiastolic dimension (LVEDd) was measured in the PLAX view from 2D registrations at maximal diameter at the site below the mitral valve. The LV endsystolic diameter (LVESd) was measured in the same view with the smallest achievable LV size. Systolic functionas regional systolic contractility was measured by SMV during the ejection phase in the same segments. Tissue tracking (TT) was used to measure longitudinal function in each segment.

Dyssynchrony was measured by 2 different methods AVO-SMV/SD (systolic maximal velocity) where the time AVO-SMV max was calculated in ms for each of the 14 segments. A mean value with a standard deviation (SD) was calculated, the SD given as a measure of dyssynchrony. We also used Tissue Synchronization Imaging (TSI). The TSI start was set at the AVO and the TSI end at the AVC in order to focus only on the systolic phase. A cut-off value of 65 ms was chosen prospectively. All the TDI analysis were performed twice by the same investigator and the mean of the two measurements was used. Intra-variability was fairly low. The coefficient of variation for TSI analysis was 3,0%.

Transthoracic 3D Echo was performed using a 2,5 MHz transducer mounted in a handheld rotation device. The cardiac images were recorded from the apex during end-expiratory apnoea within one breath hold, whereby the need for respiratory triggering was abolished. Imaging was performed using tissue harmonic mode using coaxial rotation from the apical position and ECG-triggered recording where each R-wave initiated a 30° stepwise rotation of the transducer. A total of six scanning planes obtained by 30° stepwise rotation of the transducer covered the entire LV. To eliminate disturbances caused by cardiac arrhytmias, only R-R intervals within 20% of the mean were accepted. Ejection fraction was calculated by the formula EDV-ESV/EDV × 100. Body surface area (BSA) was used to calculate EDVi and ESVi of the LV. All examinations were stored on magnetic optical discs for later off-line analysis.

### Biventricular Pacemaker Implantation

All patients received a cardiac resynchronization therapy (CRT) device (Insync III^®^, Medtronic) implanted transvenously with a lead in the right atrium and the RV lead positioned in the middle of the ventricular septum. The LV lead was positioned by identifying the sinus coronarius in the right atrium and implanted in a lateral epicardial vein in all patients except for three who had anterio-lateral position of the LV lead. The latter location was chosen due to anatomy in two patients and due to a high threshold in the lateral position in one patient.

The day after implantation the patients were examined with extensive Tissue Doppler Echocardiography (TDE). TDE was performed after 10 minutes of pacing at each VV- interval during simultaneous pacing LV lead (LV lead 4 ms before the RV lead and during sequential pacing (LV 20 and 40 ms before RV and RV 20 and 40 ms before LV lead pacing). After this examination the atrium ventricular delay was optimized by using the longest achievable (the iterative method) left ventricular filling time [13].

### Statistical analysis

Mean values ± SD are given unless otherwise stated. For within individual changes paired parametric student's t- tests were used when variables showed normal distribution and non-parametric tests, Wilcoxon matched pairs, when not. We used multiple linear regression to explain the differences in LV-endsystolic diameter and in turn as dependent variables age, gender, etiology, QRS-duration, LV lead location, SMV, TT, AVO-SMV/SD and TSI were used as possible predictors. Instead of using a step-wise method for finding the best combination of predictors we used the method of best subsets. The criterion used for ranking those subsets was "Mallows Cp". A p value < 0.05 was considered statistically significant.

## Results

### Systolic function

**Figure 1:**
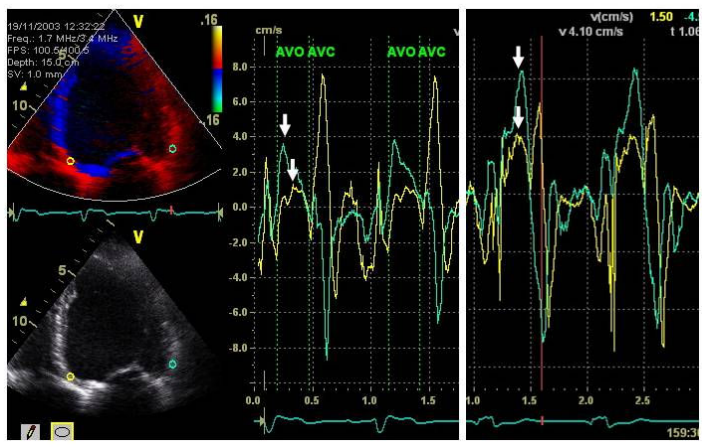
**Tissue Doppler Imaging curves with systolic maximal velocity (SMV) preoperatively (*left*) and after implantation of CRT device (*right*), at best VV-interval (sequential pacing LV lead 40 ms before RV lead in this patient), showing improved systolic function, i.e. higher velocity**. Signs of less dyssynchrony (arrows) are also seen.

**Figure 2:**
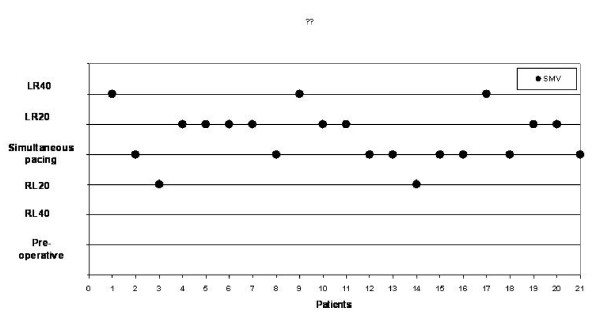
**The best VV-interval after optimization in each patient in this study (n = 21)**. With sequential pacing highest systolic maximal velocity (SMV) value was reached in 13 patients (62%).

Preoperative SMV was 3.0 ± 0.7 and at simultaneous pacing it was 3.5 ± 0, 8 cm/sec (p < 0, 01)(+18 ± 17%). At best VV-interval (sequential pacing) it increased to 3.6 ± 0,8 (p < 0,0001) an increase of 23 ± 20% (range 0.5-70.1%)(Fig 1). Compared to preoperative values, SMV was higher in 16 patients at simultaneously pacing, unchanged in one patient and lower in four patients. At the best VV-interval 17 patients had higher SMV and four patients unchanged compared to preoperative SMV value. Fourteen patients (66%) had higher SMV after VV-optimization compared with simultaneous pacing (Fig 2). Best achievable VV-interval was found with LV preactivated in 11 patients (52%), RV preactivated in two and in the remaining at simultaneous pacing (Fig 2).

**Figure 3:**
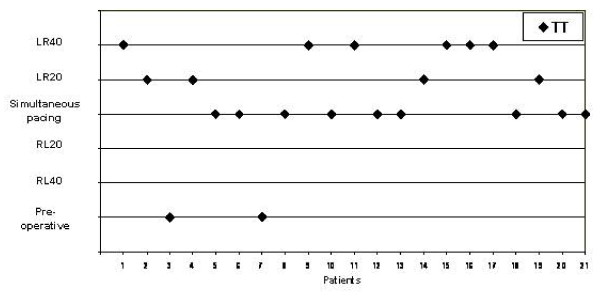
**The best VV-interval after optimization in each patient in this study (n = 21)**. With sequential pacing greatest longitudinal motion measured by tissue tracking (TT) was reached in 12 patients (57%).

Systolic function by the means of longitudinal shortening was measured by tissue tracking (TT) which preoperatively was 4.2 ± 1,5 mm and increased to 5.0 ± 1.2 mm (P < 0.05)(+29 ± 47%) at simultaneous pacing. At the best VV-interval it increased further to 5.4 ± 1.2 mm (p < 0.001) (+40 ± 53% with an individual range of -16-187%) compared to preoperative values, TT was better in 16 patients at simultaneously pacing compared with preoperative value. Twelve patients (57%) had still higher TT after VV-optimization compared with simultaneous pacing (Fig 3). There was a correlation between changes in SMV and TT (r = 0. 80, p < 0.001). However, highest SMV and TT were reached at simultaneous pacing in 8 and 9 patients, respectively.

### Dyssynchrony

**Figure 4:**
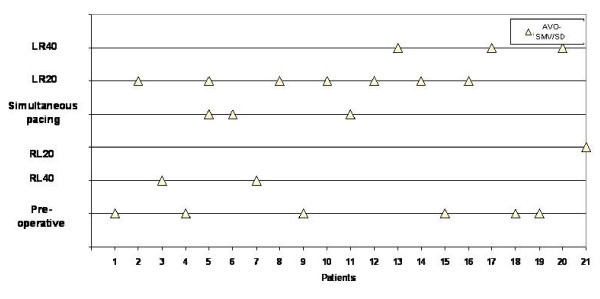
**The best VV-interval after optimization in each patient in this study (n = 21)**. With sequential pacing least dyssynchrony standard deviation of aortic valve opening to systolic maximal velocity (AVO-SMV/SD) was reached in 13 patients (62%).

Dyssynchrony measured by AVO-SMV/SD was preoperatively 40.9 ± 19.7 ms and at simultaneous pacing it tended to increase, 42.9 ± 15.0 (n.s). However, at best VV-interval it decreased to 35.0 ± 19.5 ms (P < 0,01) compared with simultaneous pacing. Compared to preoperative values, AVO-SMV/SD was better (lower) in 8 patients. Eighteen patients (86%) had less dyssynchrony after VV-optimization compared with simultaneous pacing (Fig 4).

**Figure 5:**
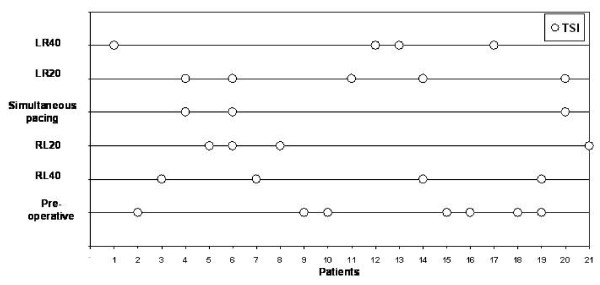
**The best VV-interval after optimization in each patient in this study (n = 21)**. With sequential pacing least dyssynchrony measured by Tissue Synchronization Imaging (TSI) was reached in 17 patients (81%).

Preoperative TSI showed dyssynchrony in 4.8 ± 2.8 segments and at simultaneous pacing in 5.9 ± 2.0 segments (p = 0,10). After VV-optimization there were 3.6 ± 2.3 affected segments (p = 0,08 compared with baseline) and statistically significantly less compared with simultaneous pacing (p < 0.001). Compared to preoperative values, TSI was better in 8 patients at simultaneously pacing and increased in 10 patients. At the best VV-interval, 16 patients (76%) had less dyssynchrony after VV-optimization compared with simultaneous pacing (Fig 5). TSI correlated with dyssynchrony measured by AVO-SMV/SD preoperatively and at best VV-interval, r = 0.48 p = 0.03 and r = 0.55 p = 0.01, respectively, but was not correlated with SMV or TT at the best VV-interval.

All variables taken together optimal pacing was achieved by sequential pacing in 60% of the patients. Looking at sequential pacing, LV before RV was best in 75% and in this group LV 20 ms before RV was best in 60%.

### Two years Follow-up

After two years 4 patients had died and 2 patients did not agree to additional examination. Therefore reevaluation of dimensions, volumes and EF could only be carried out in 15 patients. However, LVEDd decreased 7.1 mm to 62.0 ± 8.3 mm (p < 0.05) and LVESd decreased 7.3 mm to 53.0 ± 9.5 mm (p < 0.05). LVEDVi and LVESVi decreased 27.1 and 27.6 ml respectively, however not statistically significant. EF changed 4.8% to 30.8 ± 9.4%, n.s. Preoperative values are given in Table 2.

**Table 2:** Echocardiography and Tissue Doppler before device implantation (n = 21).

Variable (range)	Preoperative
Tissue Tracking, mm	4,2 ± 1,5 (2,2-6,9)
Systolic Maximal Velocity, cm/s	3,0 ± 0,7 (1,8-4,6)
AVO - SMV/SD, ms	40.9 ± 19.7 (19.7-107.2)
Tissue Synchronization Imaging, segments	4,8 ± 2,8 (0,9-8,0)
LVEDd, mm	69.1 ± 9.6 (54.8-88.7)
LVESd, mm	60,5 ± 11.2 (37,9-81,3)
EF, %	26,0 ± 7.5 (10,4-35,4)
LVEDVi, ml/m^2^	128,4 ± 51,6 (63,1-234,3)
LVESVi, ml/m^2^	100,2 ± 47,2 (57,3-202,0)

Mean ± SD. AVO Aortic Valve Opening, SMV = Systolic Maximal Velocity, LVEDd = left ventricular end diastolic diameter, LVESd = left ventricular end systolic diameter, EF = ejection fraction. EDVi = end diastolic volume index, ESVi = end systolic volume index.

A multiple linear regression analysis was performed to see which variables explained the changes in LV dimensions. Examined variables were age, gender, etiology, QRS-duration, LV lead location, SMV, TT, AVO-SMV/SD and TSI. The only variables explaining changes in LV dimensions were SMV, AVO-SMV/SD and TSI.

## Discussion

The main finding in this study is that approximately 2 of 3 patients with chronic heart failure (CHF) receiving cardiac resynchronization therapy (CRT) improve their systolic heart function in the acute phase more with sequential biventricular pacing than with simultaneous biventricular pacing. This when systolic function is expressed as regional contractility, longitudinal motion and dyssynchrony measured by Tissue Doppler Imaging echocardiography. Choosing sequential pacing, the best choice in this study turned out to be pacing the LV lead before the RV lead and of tested programming LV lead 20 ms before RV lead turned out to have best effect on systolic function.

Bearing the serious prognosis of these HF patients in mind it should be obvious trying to get as much beneficial effect as possible from the CRT device through individual optimization. The highest individual increase in this study was 187% of the TT value (sequential pacing) compared with 75% at simultaneous pacing. Similar results have been achieved by others [14].

An important question is whether to VV-optimize, AV-delay optimize or to do both or to do neither.

It's difficult to say for sure what is right since there today are too few long-term randomized controlled trials (RCT's) to give a clear answer to this question. There are mainly three types of dyssychrony which are to be treated with CRT; inter-ventricular and intra-ventricular (both affected by VV optimization) and atrio-ventricular (affected by AV-delay optimization). The inter-ventricular mechanical delay (IVMD), is defined as the time difference between the onset of forward flow in the LV outflow tract and RV outflow tract >40 ms and intra-ventricular as when the time difference between regional myocardial maximal velocities exceed 65 ms. Atrio-ventricular dyssynchrony (LVFT/R-R) is considered present if the left ventricular filling time (LVFT) measured from the mitral valve Doppler in-flow signal is <40% of the cardiac interval (R-R). The usual proportion of these different types of dyssynchrony are 60%, 80% and 30% in such HF patients with reduced ejection fraction and prolonged QRS [15-18]. Moreover, the relationship between these types of dyssynchrony is reported to be poor.

Thus, the figures presented in this study strongly suggest an individualized approach to optimization of the CRT device since some patients might benefit from VV-optimization, others from AV-delay optimization and others from both. How reverse remodelling influences on dyssynchrony and long-term effects of device optimization should be studied in future RCT's. Two published RCT's have however not demonstrated any beneficial effects of VV optimization on LV dimensions, functional class or QoL [19,20].

There are many ways to measure hemodynamik effects of device programming.

**Figure 6:**
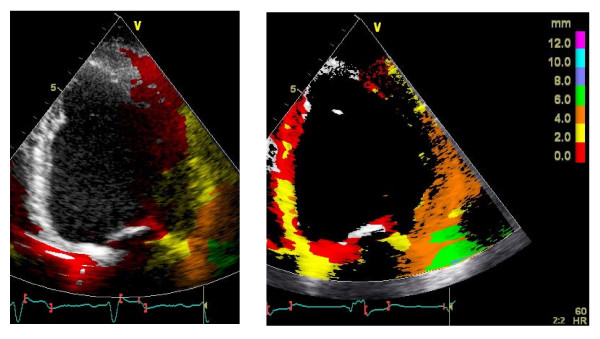
**Tissue tracking (TT) preoperatively (*left*) and after implantation of CRT device (*right*), at best VV-interval (sequential pacing LV lead 20 ms before RV lead in this patient), showing improved systolic function, i.e. longitudinal motion**. TT is a fast non-invasive method to study changes in systolic function fairly close related with changes in SMV (r = 0.80, p < 0.001).

Echocardiography measuring stroke volume by left ventricular outflow tract (LVOT) VTI is perhaps most used. We prefer the TDI method, since we are used to it and therefore find it a time efficient method to find the most appropriate programming with regard to it's relation with regional contractility (dP/dt) and longitudinal function. This can also be achieved by visual examination only, without the need for measurements (Fig 6). Thus, a fast non-invasive method. Invasive dP/dt, blood pressure, finger plethysmography, cardiac impedance, electrocardiogram and intracardiac electrogram (IEGM) are all useful methods, at least in the acute phase, but they will probably show slightly different results. Most important is however, to use methods which one is accosted to and relies on.

### Study limitations

A major limitation of this study is the lack of randomization and the small patient number. Designing this trial again we would have included more patients and performed a randomization to assess the long-term effects of VV-optimization added onAV-delay optimization on LV function, NYHA-class and QoL. However, we believe this pilot study is important for highlighting the potential individual effect of VV-optimization in HF patients with prolonged QRS. Thus, even if this study is small and contains no long-term data, VV-optimization the first day after CRT implantation the study highlights the potential benefits of such strategy.

The PROSPECT (Predictors of response to cardiac resynchronization therapy) trial, an observational study, was somewhat disappointing concerning the value of measuring dyssynchrony [21]. This was a multicentre study including 426 patients with standard indication for CRT. The aim was to identify echocardiography and tissue Doppler measures of dyssynchrony and their ability to predict response to CRT. It was found that presence of signs of dyssynchrony was linked to 11-13% additional clinical response to CRT and 13-23% additional response for reverse remodelling compared to absence of measures of dyssynchrony. The main reason was a high inter-variability measuring signs of dyssynchrony. However, in a recently published sub analysis of this trial dyssynchrony was pointed out as one of the baseline characteristics to benefit of biventricular pacing [22]. The most useful measures of dyssynchrony according to this analysis are inter-ventricular (IVMD) and intra-ventricular (Ts lat-sept) measures because these are the measures with lowest inter-variability. The analysis also points out other important baseline characteristics for being a responder to CRT as female gender, non-ischemic ethiology, NYHA-class III, QRS-duration and having no history of ventricular tachycardia. These results show the difficulty of predicting a responder and would rather support our hypothesis that optimization of the device could be important during the first months before the remodelling process takes place.

Thus it is important to perform a solid echocardiography examination not only to assess LV systolic function and degree of dyssynchrony but also to avoid other causes of heart failure, which shouldn't be treated with biventricular pacing, as valve diseases and non-viable myocardium. The latter can be done by a low-dose dobutamin stress-echocardiography examination.

## Conclusions

Approximately 2 of 3 patients with chronic heart failure receiving cardiac resynchronization therapy improve their systolic heart function in the acute phase more with sequential biventricular pacing than with simultaneous biventricular pacing. Choosing sequential pacing, the best choice is to pace the LV lead before the RV lead and of tested programming LV lead 20 ms before RV lead turned out to have best effect on systolic function. The long-term clinical importance of these findings should be studied further.

## Funding

This study was supported with grants from the Karolinska Institutet and Medtronic.

## Conflict of interests

ME has received honoraria and research support from Medtronic.

MR and TS have no conflicts of interest to declare.

## Authors' contributions

ME: Made substantial contributions to conception and design, acquisition, analysis and interpretation of data, mainly responsible for drafting the manuscript.

MR: acquisition, analysis and interpretation of data. TS: acquisition, analysis and interpretation of data. All authors have read and approved the final manuscript.
